# Psychological intervention based on cognitive behavioral therapy for patients with orthopedic surgical anxiety

**DOI:** 10.1097/MD.0000000000039401

**Published:** 2024-08-30

**Authors:** Hui Han, Chunhua Chen, Rong Sheng, Shiying Wang

**Affiliations:** a Outpatient Department, 905th Chinese People’s Liberation Army Navy Hospital, Shanghai, China; b Health Management Center, Second Affiliated Hospital of Navy Military Medical University, Shanghai, China; c Outpatient Department, Second Affiliated Hospital of Navy Military Medical University, Shanghai, China; d Disinfection Supply Center, Second Affiliated Hospital of Navy Military Medical University, Shanghai, China.

**Keywords:** cognitive behavioral therapy, orthopedic surgery, psychological interventions, questionnaire method, statistics

## Abstract

To develop a set of cognitive behavioral therapies (CBTs) to alleviate anxiety in orthopedic surgery (OS) patients, to explore the intervention effects of CBTs on the indicators of anxiety, sleep quality, and pain sensation in OS patients, and to promote them. A total of 68 qualified subjects were selected from among the 103 patients with orthopedic diseases who were hospitalized in the orthopedic department of the hospital between June 2022 and November 2023. According to the different nursing methods, they were divided into a psychological intervention (PI) group and a control intervention group. Among them, 34 patients received hospital-developed CBT for OS in the PI group, and 34 patients received standard orders from the medical staff in the control intervention group. Tools such as self-assessment of anxiety, Athens insomnia scale, state anxiety scale, visual analog pain method, and self-management level scale were utilized to assess the change in anxiety levels, sleep quality, pain perception, and self-management level of the 2 groups of patients before and after the surgery. Following the CBT intervention, patients in the PI group had significantly lower Athens insomnia scale (5.32 ± 0.42), state anxiety scale (38.21 ± 1.12), and visual analog pain method (3.93 ± 1.24) scores than those in the control intervention group. This difference was statistically significant (*P* < .05). In the meantime, patients in the PI group had a substantially higher correct rate of illness cognition assessment (98.21%) than patients in the control intervention group (65.12%), and this difference was statistically significant (*P* < .05). The study collated the factors affecting anxiety in OS patients through questionnaire survey and statistical analysis experiment and then formulated a detailed CBT strategy for specific problems. Finally, CBT is a valuable tool for reducing anxiety in OS patients. As such, it deserves to be promoted and used in clinical settings.

## 1. Introduction

Patients frequently experience anxiety related to orthopedic surgery (OS), which can have a detrimental impact on the patient’s recovery after surgery in addition to making the procedure go more smoothly.^[1]^ Therefore, appropriate psychological intervention (PI) measures are important to alleviate OS anxiety. Currently, the treatment of anxiety and other adverse emotions is mainly based on medication, supplemented by PI. Medication has great side effects and is only effective for positive symptoms, while the traditional PI approach is relatively homogenous and focuses mainly on pain management and physical function recovery, neglecting the patient’s psychological condition and anxiety.^[2]^ Cognitive behavioral therapy (CBT), on the other hand, focuses on both the individual’s thinking process and behavioral patterns to alleviate anxiety symptoms through cognitive restructuring and behavioral changes. It not only helps patients to understand and adjust negative thinking patterns but also encourages them to develop positive thinking styles and adaptive behavioral patterns and has been gradually applied in clinical research in recent years.^[3,4]^ This method can help correct patients’ misperceptions before surgery, improve patients’ adverse emotions and behaviors, and improve the success rate of postoperative rehabilitation. However, the protocol of cognitive therapy is not yet standardized, and the study conducted a questionnaire survey and statistical analysis based on the actual situation of the patients, looking forward to the development of a set of cognitive behavioral PI programs suitable for improving the anxiety of OS patients.

## 2. Information and methods

### 2.1. General information

One hundred three cases of patients with orthopedic diseases hospitalized in the orthopedic department of the hospital from June 2022 to November 2023 were selected to receive and collect the questionnaire survey of the patients and the corresponding treatment data of orthopedic diseases for the study. Inclusion criteria: patients selected for OS treatment and conventional conservative treatment in the hospital orthopedic department; adults with normal mental status, clear consciousness, and other healthy physiological conditions who can cooperate with the successful completion of the experiment; all medical records were complete; the patient has not received other forms of treatment; and the patients have signed an informed agreement to participate in this experimental study on a voluntary basis. Exclusion criteria: individuals with substantial medical experience, psychotherapy, or a history of mental illness and patients who are unable to accurately describe their feelings.

The patients and their families completed an informed consent form; the study was approved by the medical ethics committee.

### 2.2. Research methods

#### 2.2.1. Questionnaire method

Patients’ state anxiety was initially assessed using the Spielberger scholars’ “state anxiety scale” (SAS), where a greater score indicated a higher degree of preoperative anxiety.^[5]^ The patients’ anxiety was then assessed using the self-assessment of anxiety (SAI), which was created by Zung academics. The higher the patient’s score, the more anxious they were before surgery.^[6]^ Then, the degree of insomnia of the patients was assessed using the Athens insomnia scale (AIS) developed by Prof Charles Morin.^[7]^ Finally, the visual analog pain method (VAS) created by scholars Stewart G.W. Mercer and Robert Melzack was used to assess the patient’s pain sensation.^[8]^ Before the questionnaire survey, each patient should get a personal explanation of its contents, and the assessors should be qualified to conduct assessments in order to guarantee the correctness of the test results. After the test, 2 healthcare workers collected, organized, and analyzed the data, excluded incomplete and invalid questionnaires, and entered the qualified questionnaire results into the analysis system.

#### 2.2.2. CBT on orthopedic patient’s preoperative anxiety PI method design

The PI program was developed based on the main factors affecting the patient’s anxiety in combination with CBT as derived from the above program measurements. The CBT-based PI strategies developed were divided into cognitive strategy intervention and behavioral strategy intervention. The steps of cognitive strategy intervention are given as follows. At the time of patient admission, provide health education, correct misconceptions or perceptions, provide correct information and understanding of the condition, and guide the patient to familiarize with the hospital inpatient and surgical environments. Before surgery, explain the steps and precautions of the surgical procedure to help patients prepare and eliminate anxiety before surgery. On the day after surgery, patients are taught techniques for coping with pain and provided with specific ways to do so. Share tips and advice on improving sleep quality to help patients improve their sleep quality after surgery. Prior to discharge, patients are instructed to focus on postillness recovery, precautions, and self-management to increase confidence. The behavioral strategy intervention steps are given as follows. At the time of admission, lead patients to engage in positive activities and behaviors such as relaxation exercises and stretching. Prior to surgery, guide the patient through breathing and meditation to reduce anxiety and stress prior to surgery. On the day after surgery, help the patient to reduce possible pain after surgery through progressive muscle relaxation. Also, guide patients in abdominal breathing techniques to promote relaxation and improve sleep quality. Before discharge from the hospital, provide advice on ways to return to exercise and healthy lifestyle habits.

**Figure 1.**
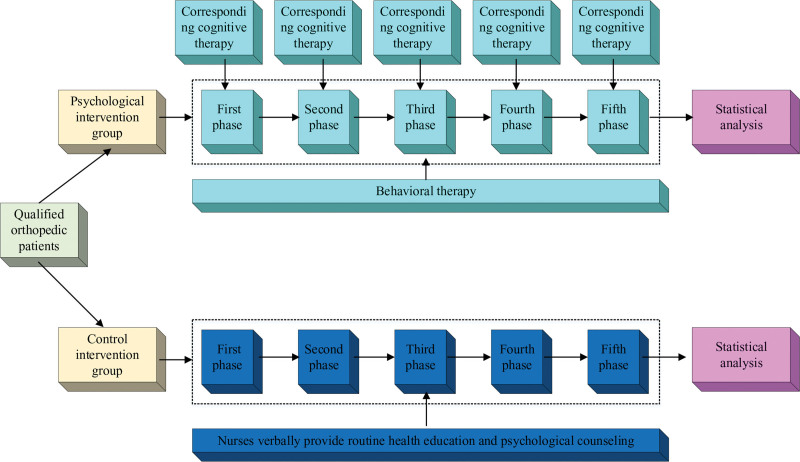
Experimental flow of cognitive behavioral therapy–based psychological interventions.

The study divided the patients belonging to OS anxiety patients (i.e., SAS50 score) in the above assessment into the PI group and control intervention group depending on the type of care (Fig. 1). In the control intervention group, the treatment is given as follows: healthcare professionals followed the conventional healthcare measures and only verbally gave the patients routine health education and psychological counseling. The PI group was treated as follows: CBT-based adjunctive interventions were provided to patients by physicians who had studied CBT and were qualified. The half-hour interventions took place at the time of hospitalization, 3 days before surgery, 1 day after surgery, 3 days following surgery, and 1 or 2 days before release.

In the specifics of cognitive therapy, in the first stage, the medical staff explains the knowledge of anxiety and treatment methods and knowledge related to the disease. In the second stage, the medical staff explains the entire surgical procedure and the environment in which the surgery is performed. In the third stage, pain management methods and postoperative precautions are explained. In the fourth stage, listening, comforting, evaluating, and giving positive guidance are combined with the sharing of relevant past successful case program approaches to provide patients with positive and healthy psychological support to alleviate anxiety. The fifth stage explains postdischarge medication, follow-up, and nursing health education to help patients develop a healthy and positive outlook on life. For the specifics of behavioral therapy, the medical staff guided the patient through relaxation exercises twice daily, including intentional relaxation exercises, tension relaxation exercises, meditative counting breath training, sleep relaxation exercises, and meditative body scanning. The hours were 7:00 to 7:30 am and 10:00 to 10:30 pm. Intentional relaxation exercises. The patient is guided to close his/her eyes and focus on the intentional relaxation of each muscle group, starting from the head and gradually relaxing down to the feet. When relaxing in a certain area, the patient consciously focuses on that area, feels the sensation of relaxation, and imagines that he or she is gradually sinking into a gentle, warm atmosphere. Tension relaxation exercise: have the patient consciously contract a muscle group, hold it for a few seconds, and then release it to allow the muscle group to completely relax and repeat the process. Meditation counting training: guide the patient to take a deep breath and count the number of exhalations and inhalations, as a way to adjust the rhythm and depth of breathing and reduce anxiety. Sleep relaxation exercises: relaxation exercises are performed before bedtime. Through soothing music and breathing exercises, patients are gradually sedated and prepared to enter a state of deep relaxation. Meditative body scanning: guide patients to close their eyes and notice and feel the sensations of the body part by part, starting from the head and gradually scanning to the feet, staying for a moment in each part to focus on the sensations and relaxation as a way to deepen the connection between the body and consciousness.

#### 2.2.3. Measurement of outcome indicators

The assessment tools were the anxiety self-assessment scale, AIS, visual analog scale, and self-management level assessment scale. The patients underwent daily testing throughout the experimental period to assess their cognitive abilities in relation to orthopedic diseases. The test covered the knowledge of the diseases as explained by the hospital’s doctors, and it took the form of a closed-book question and answer session.

#### 2.2.4. Statistical analysis method

The selected software for data analysis was SPSS 23.0 statistical software. Different statistical methods were used to analyze different test indicators. Single factors affecting patients’ anxiety status were analyzed using *t* test and ANOVA. Anxiety and pain, as well as anxiety and sleep, were examined using Pearson correlation analysis. Three or more participants’ influencing factors were examined using multiple linear regression analysis. Frequency counts were used to describe the general information of the patients.

## 3. Results

### 3.1. Results of preoperative anxiety state measurement in orthopedic patients

**Figure 2.**
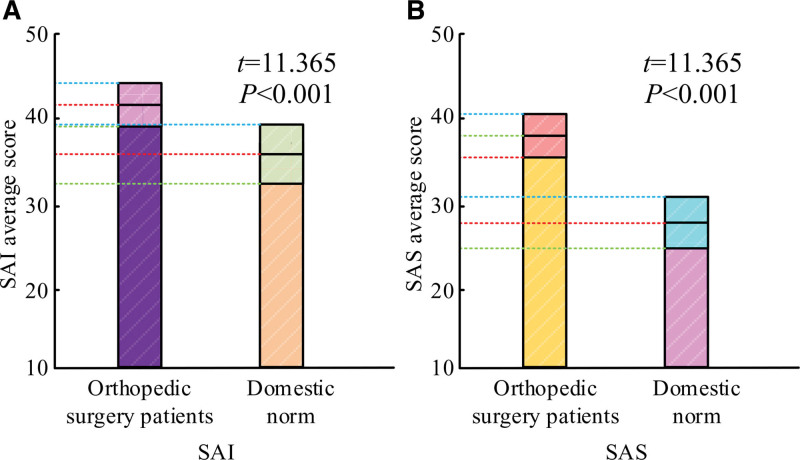
Comparison of preoperative anxiety state scores of orthopedic patients with national norms. SAI = self-assessment of anxiety, SAS = state anxiety scale.

The results of the anxiety state scores of 103 patients in the hospital and the scores of 960 domestic norms (a set of domestic reference standards for preoperative state anxiety scores of OS patients) are shown in Figure 2A. In this figure, the preoperative SAI scores of orthopedic patients were 42.32 ± 4.36, whereas the scores of the domestic norms were 36.21 ± 6.32. In Figure 2B, orthopedic patients’ presurgery SAS score was 38.12 ± 3.65, while the score of the domestic norm was 29.01 ± 5.01, which indicates that orthopedic patients in hospitals are generally more anxious and tense before surgery.

### 3.2. Analysis of the correlation between prehand anxiety scores and sleep quality and pain perception in orthopedic patients

**Figure 3.**
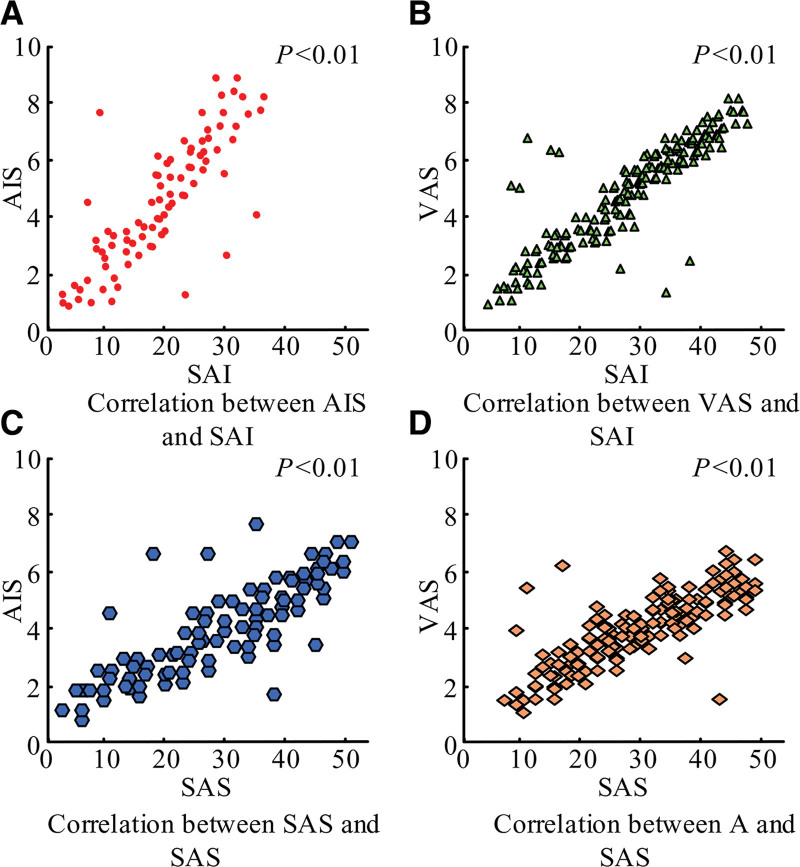
Results of the correlation analysis of preoperative anxiety state with sleep and pain in orthopedic patients. SAI = self-assessment of anxiety, SAS = state anxiety scale.

The results of the correlation between SAI score and SAS score with AIS score and VAS score of orthopedic patients are shown in Figure 3. In this figure, there was a positive correlation between SAI and AIS, SAI and VAS, SAS and AIS, and SAS and VAS with correlation coefficients of 0.732, 0.695, 0.423, and 0.421, respectively. It indicated that the more anxious and tense the patients were before the surgery, the sleep quality decreased, and pain increased. Similarly, decreased sleep quality contributes to excessive anxiety and tension in patients.

### 3.3. Factors affecting preoperative anxiety scores in orthopedic patients

Multiple linear regression analysis was utilized to assess the SAS and SAI scores as dependent variables and the statistically significant influencing factors as independent variables.

As shown in Table 1, the main factors affecting anxiety in orthopedic patients were sleep quality, pain, perception of the disease, and the presence or absence of surgical experience.

**Table 1:** Results of multiple linear regression analysis of factors affecting preoperative anxiety scores in orthopedic patients.

	Regression coefficient	Standard error	*t* values	*P* values
Multivariate stepwise regression analysis of factors influencing preoperative SAI scores in orthopedic patients				
Sleep quality	2.135	1.021	16.32	<.001
Pain sensation	0.458	0.124	5.634	<.001
Industry engaged in	0.963	0.224	0.163	<.001
Cognition of diseases	0.712	0.123	2.653	.015
Disease type	−0.912	0.312	−0.386	<.001
Have you had any surgical experience?	2.635	0.512	4.635	<.001
Multivariate stepwise regression analysis of factors influencing preoperative SAS scores in orthopedic patients				
Gender	2.365	0.763	3.645	<.001
Age, yr	1.365	0.456	3.124	.004
Sleep quality	0.963	0.247	4.698	<.001
Pain sensation	0.634	0.124	2.865	.005
Cognition of diseases	0.841	0.345	2.687	.032

SAI = self-assessment of anxiety, SAS = state anxiety scale.

### 3.4. Comparison of information on orthopedic patients participating in CBT intervention trials

Of the 103 orthopedic patients who fulfilled the eligibility criteria for the PI trial of CBT, 68 were split equally into 2 groups: 34 patients were assigned to the PI group and 34 patients were assigned to the control intervention group. Table 2 displays the data for the 68 orthopedic patients.

**Table 2:** Comparison of patient information between the psychological interventions group and the control intervention group.

		Psychological intervention group (34 members)	Control intervention group (34 individuals)	Statistic
Sex ratio		16:18	17:17	X^2^ = 0.324
Age, yr		46.12 ± 1.36	45.42 ± 2.11	X^2^ = 0.218
Engage in profession	Freelance	14	13	X^2^ = 0.765
	Teachers, doctors, civil servants, etc	7	8	
	Farmer	9	8	
	No job	4	5	
Have you had any surgical experience?	Yes	9	10	X^2^ = 0.498
	No	25	24	
Do you have any understanding of the disease?	Yes	5	4	X^2^ = 0.312
	No	29	30	
SAS		56.34 ± 1.32	56.12 ± 1.45	X^2^ = 0.563
AIS		8.96 ± 1.63	9.12 ± 1.24	X^2^ = 0.247
VAS		6.41 ± 1.41	6.39 ± 1.52	X^2^ = 0.614

AIS = Athens insomnia scale, SAS = state anxiety scale, VAS = visual analog pain method.

Table 2 shows that there was no significant difference in the SAS, AIS, VAS, gender, age, occupation, surgical experience, or awareness of the condition between the 2 patient groups that took part in the experiment (*P* > .05).

### 3.5. SAS scores, AIS scores, and VAS scores of orthopedic patients participating in a CBT intervention trial

**Figure 4.**
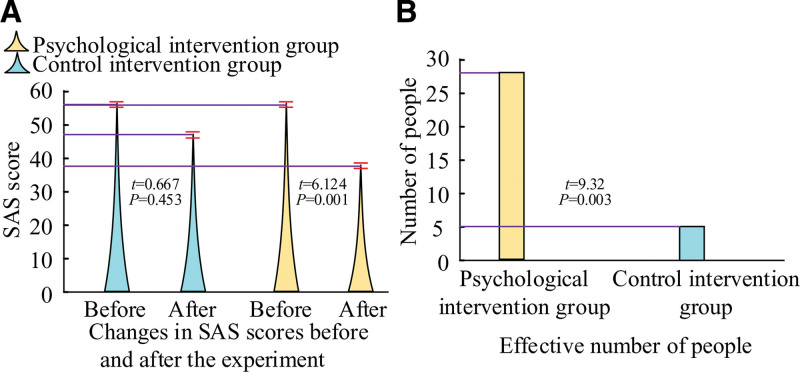
Results of state anxiety scale (SAS) scores before and after the experiment for both groups of patients.

Figure 4 displays the SAS scores of the 2 patient groups following the application of CBT-based PI.

Following the trial, the patients in the PI group had a decrease in their SAS scores from 56.341.32 to 38.211.12, while the patients in the control intervention group saw a decrease in their SAS scores from 56.121.45 to 47.961.38 (Fig. 4A). The patients who received CBT-based adjunctive interventions showed a significant reduction in anxiety and a lower final score than the control intervention group, indicating that CBT’s study design has a greater impact on lowering anxiety levels. There were 5 successful interventions in the control intervention group and 28 in the PI group, as shown in Figure 4B. It also further indicates that the program designed in the study has a significant effect.

**Figure 5.**
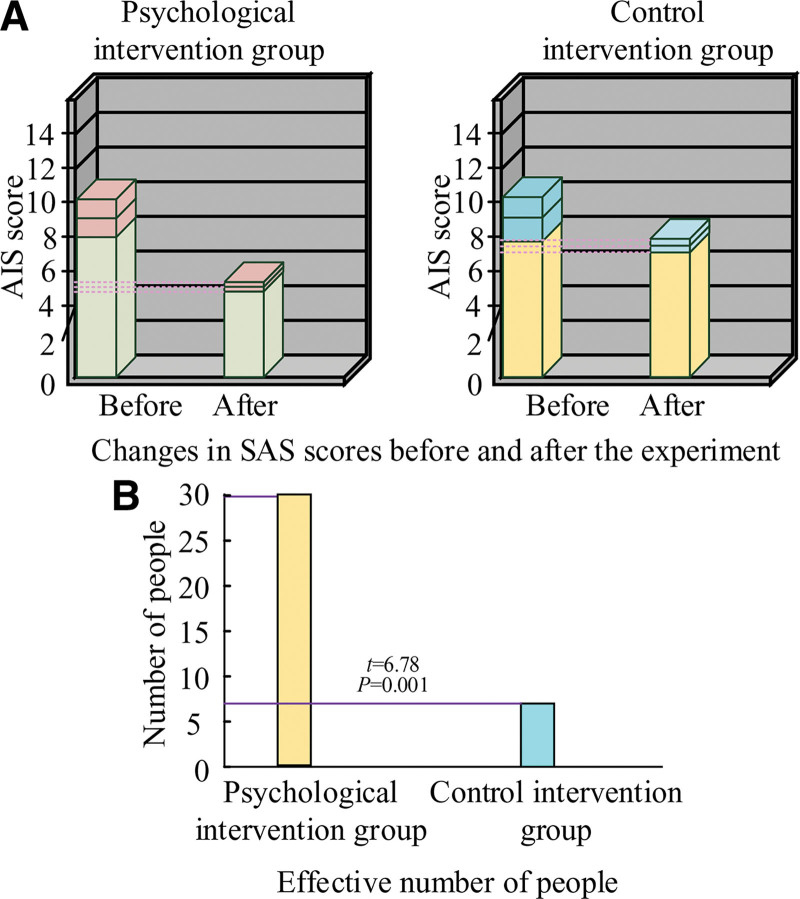
Results of Athens insomnia scale (AIS) scores before and after the experiment for both groups of patients. SAS = state anxiety scale.

The AIS scores of the patients in both groups are shown in Figure 5. Following the trial, patients in the PI group had a decrease in AIS scores from 8.96 ± 1.63 to 5.32 ± 0.42, while patients in the control intervention group saw a decrease in AIS scores from 9.12 ± 1.24 to 7.45 ± 0.32, as shown in Figure 5A. Conclusion: the patients in the PI group showed a stronger tendency toward decline in their AIS scores, and their final score was lower than the control intervention group’s. This suggests that the CBT used in the study had a higher impact on enhancing the quality of postoperative sleep. Thirty were effective improvements in the PI group and 7 in the control intervention group, as shown in Figure 5B. It further illustrates that the program designed in the study was effective in improving the sleep of the patients.

**Figure 6.**
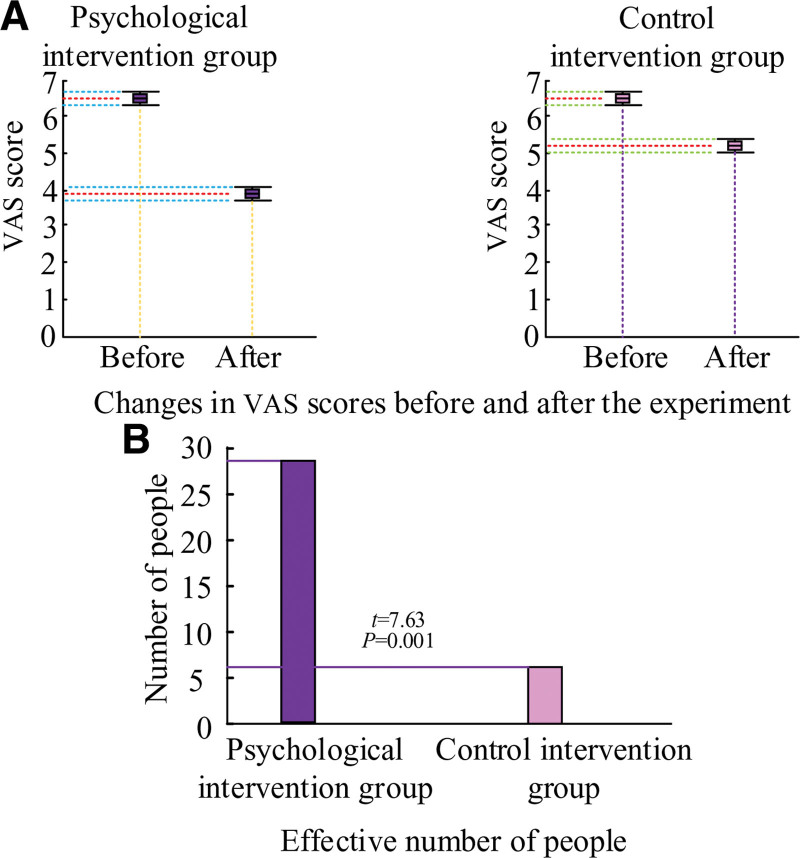
Results of visual analog pain method (VAS) scores before and after the experiment for both groups of patients.

The VAS scores of the patients in both groups are shown in Figure 6. Following the trial, patients in the PI group had a decrease in their AIS scores from 6.41 ± 1.41 to 3.93 ± 1.24, while patients in the control intervention group saw a decrease in their AIS scores from 6.39 ± 1.52 to 5.22 ± 1.35, as shown in Figure 6A. The PI group’s patients’ AIS scores decreased more than those of the control intervention group’s patients, and the PI group’s final score was lower than the control group’s, suggesting that the CBT used in the study had a more substantial impact on participants’ perceptions of pain. As illustrated in Figure 6B, there were 29 successful improvements in the PI group and only 6 successful interventions in the control intervention group.

### 3.6. Disease perceptions in orthopedic patients participating in a CBT intervention trial

**Figure 7.**
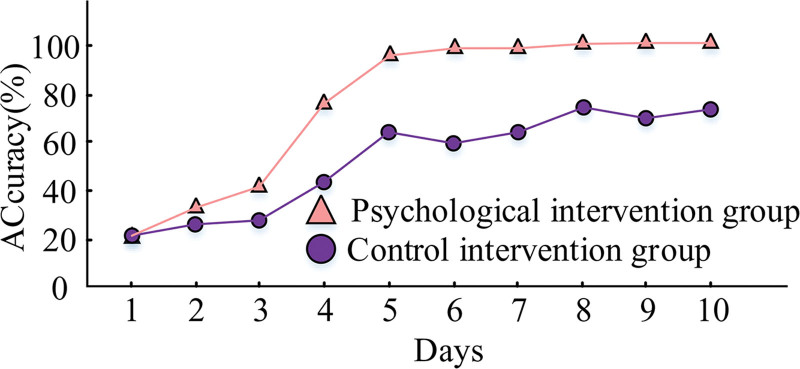
Results of patients’ disease–related cognitive tests.

Every day, both orthopedic patient groups received disease-related cognition assessments in the form of a closed-book examination. Figure 7 displays the results.

In Figure 7, when they were first admitted to the hospital, the correct rate of both groups was low, only 20.21%. However, as the time of admission increased, the medical staff used different ways to explain the cognitive knowledge of related diseases to the patients in both groups. Patients in the PI group experienced a dramatic increase in the correct answer rate on days 3 and 5, which persisted above 98.21%. The correct answer rate of patients in both groups started to rise. Throughout the course of the intervention, the patients in the control group saw fluctuations in their accurate answer rate, which eventually reached approximately 65.12%. This indicates that the methodology used in the study was effective in increasing the patients’ correct knowledge of the disease and had a reinforcing effect that could be maintained over time.

### 3.7. Self-management efficacy in orthopedic patients participating in a CBT intervention trial

Table 3 displays a comparison of the orthopedic patients’ self-management scores from the 2 groups before and after the intervention.

**Table 3:** Results of self-management scores of patients in both groups.

		Dietary action	Physical activity	Social psychological behavior	Treatment behavior	Total score
Psychological intervention group	Before intervention	21.63 ± 1.25	9.65 ± 2.21	10.11 ± 1.21	26.38 ± 0.96	78.64 ± 3.65
	After intervention	35.54 ± 1.65	24.65 ± 1.96	47.34 ± 1.56	49.63 ± 1.21	91.32 ± 2.54
Control intervention group	Before intervention	23.11 ± 1.02	10.12 ± 1.96	9.12 ± 1.96	25.1 ± 3.96	79.11 ± 2.65
	After intervention	25.43 ± 1.34	11.65 ± 1.85	11.65 ± 1.85	26.34 ± 1.32	84.63 ± 3.15
*t*_1_		7.11	3.65	2.336	1.25	1.78
*t*_2_		7.65*	7.01*	9.54*	6.35*	9.35*
*t*_3_		6.24*	5.63*	9.12*	4.68*	8.65*
*t*_4_		9.21	5.12	6.93	7.63	5.63

Note: *t*_1_ is the between-group comparison between the 2 groups, *t*_2_ is the between-group comparison between the 2 groups after the intervention, *t*_3_ is the comparison between the psychological intervention group itself before and after the intervention, and *t*_4_ is the comparison between the control intervention group itself before and after the intervention.

* indicates *P* < 0.05 is statistically significant.

In Table 3, before the experimental intervention, the scores of patients in each group on eating actions, somatic activities, psychosocial behaviors, and therapeutic behaviors were not significantly different from each other. Following the psycho-cognitive program intervention, the PI group’s scores significantly improved in all areas, with their overall self-management score rising from 78.64 ± 3.65 to 91.32 ± 2.54. In contrast, the control group’s self-management level only saw a slight increase, from 79.11 ± 2.65 to 84.63 ± 3.15, and the difference was statistically significant in each case. It is evident that the patient’s degree of self-management significantly improved as a result of the study’s application of CBT.

## 4. Discussion

### 4.1. Orthopedic patients have high levels of anxiety about surgery

The study’s findings demonstrated that orthopedic patients’ presurgical SAI scores were 42.32 ± 4.36 points, as opposed to the 36.21 ± 6.32 points of the national standard. The SAS score was 38.12 ± 3.65, while the score of the national norm was 29.01 ± 5.01. The anxiety scores of both were higher than the domestic norm, indicating that at this stage, the anxiety level of orthopedic patients about surgery is generally high in China. This was consistent with the results of Allard et al^[9]^ who assessed patients’ anxiety levels about diseases by the psychometric method. This was due to the general perception among patients that OS usually involves greater surgical risk and complexity, which internally leads to elevated levels of anxiety out of concern about whether the surgery will be successfully completed.^[10]^ Moreover, orthopedic diseases themselves can significantly affect the patient’s life function and mobility, and this effect may exacerbate the patient’s anxiety level.^[11]^ In addition, the contrast between the personal life situation before and after the disease can cause a strong psychological stress reaction and exacerbate the patient’s anxiety level. Consequently, in order to lessen patients’ anxiety and enhance both the efficacy of surgical therapy and their mental health, healthcare providers must focus more on the mental health of their patients in their clinical work and offer the proper support and help.^[12]^

### 4.2. Preoperative anxiety factors are correlative in orthopedic patients

The study’s findings, with correlation values of 0.732, 0.695, 0.423, and 0.421, respectively, demonstrated a favorable association between SAI and AIS, SAI and VAS, SAS and AIS, and SAS and VAS. This suggested that the components had a substantial link with one another. Poor sleep quality aggravates anxiety in orthopedic patients, and anxiety leads to insomnia. This was due to the pain caused by orthopedic diseases and the change in the sleep environment, which made it difficult to ensure the quality of patients’ rest and increased their anxiety. At the same time, psychological burdens such as anxiety and worry cause patients to have difficulty falling asleep or maintaining a good sleep.^[13]^ Provide a good sleeping environment and reduce noise in the ward. Orthopedic patients, especially traumatized patients, suffer from high pain levels due to factors such as large trauma areas and restricted movement. In addition to impairing the patient’s quality of life, this discomfort also makes them more anxious, disrupts their sleep and vital signs, and causes stress, which, in turn, triggers negative emotions.^[14]^ For this reason, healthcare professionals need to pay attention to how to effectively help patients relieve pain and reduce anxiety. Therefore, when developing a program for CBT, it is important to consider all factors comprehensively.

### 4.3. CBT programs can be effective in reducing patient anxiety

Data show that anxiety leads to increased levels of stress hormones in the patient’s body, which affects immune function and, thus, slows down the wound healing process.^[15]^ Anxiety puts the body in a constant state of stress, affecting the allocation of energy and resources needed for wound healing, leading to prolonged recovery time.^[16]^ It can be concluded that anxiety can directly affect the recovery of patients. The CBT used in the study can help patients correct incorrect behaviors and misperceptions, and it was applied to OS patients. The results showed that after CBT for PI, the SAS scores of patients in the PI group were 38.21 ± 1.12, and those of patients in the control group were 47.96 ± 1.38. By comparing the patients’ SAS scores between the 2 groups, it became evident that the study’s use of cognitive behavioral treatment could significantly reduce the patient’s anxiety. This was consistent with several studies on CBT techniques to improve anxiety. Unlike the traditional single psychological therapy, the CBT adopted in the study targeted the implementation measures from both psychological and physiological aspects, saving human resources, while effectively reducing patients’ anxiety.

### 4.4. CBT program can effectively improve patients’ sleep quality and pain status

The patient’s anxiety is closely related to sleep quality and pain conditions, and anxiety affects the areas of the brain that deal with pain, enhancing the nervous system’s sensitivity to uncomfortable symptoms. Physical discomfort can cause the patient’s anxiety and fear to rise. Currently, most of the medication regimens used for patients’ symptoms of insomnia and pain are medication regimens, such as sleeping pills and nerve pain relievers. Although these drugs can briefly relieve symptoms, they ultimately have significant side effects. The treatment of nonpharmacological PI has a greater advantage, and the data show that the current psychotherapy is relatively single, such as distraction and music therapy.^[17]^ The CBT used in the study combined pain knowledge, sleep health education, and psychological techniques to help patients alleviate excessive worry, change negative cognition, provide correct information, and perform standard relaxation training to intervene in patients’ sleep and pain.^[18]^ The outcomes demonstrated that following CBT PI, patients in the PI group had AIS scores and VAS scores of 5.32 ± 0.42 and 3.93 ± 1.24, respectively, while patients in the control intervention group had AIS scores and VAS scores of 7.45 ± 0.32 and 5.22 ± 1.35, respectively. These findings suggest that CBT significantly improves patients’ pain and sleep.

### 4.5. CBT programs can improve patient self-management

The study’s findings demonstrated that patients in the PI group experienced an increase in total self-management score from 78.64 ± 3.65 to 91.32 ± 2.54 following cognitive behavioral treatment for PI, while patients in the control intervention group only saw a slight increase in total self-management from 79.11 ± 2.65 to 84.63 ± 3.15. It can be inferred that following CBT, the patient’s degree of self-management increased dramatically, which is in line with the findings of Barroso.^[18]^ The results were consistent with the results of self-management level measurement in patients with diseases^[18]^ because when a major disease occurs, people have large fluctuations in their emotions and irrational beliefs about the lack of knowledge about the disease, and such emotions and beliefs greatly affect the patients’ coping styles and self-management levels.^[19,20]^ CBT intervenes and corrects patients’ beliefs and misperceptions and guides them to adopt positive behaviors to solve problems. For patients with orthopedic diseases, improving the level of self-management is crucial for postoperative rehabilitation. Therefore, CBT can be used as an effective strategy to help orthopedic patients recover their health faster.

Although the study designed an experiment to evaluate the intervention effect of CBT on indicators of anxiety, sleep, pain, and self-management level in orthopedic patients and achieved some success, the experiment also has some limitations. First, the sample capacity is small, which limits the generalization of the results. Moreover, the selected subjects were only hospital patients, and hospital patients cannot represent all orthopedic patients, limiting the generalizability and usefulness of the results. In addition, as an observational study, the experimental results are inevitably subject to chance and bias. Therefore, large-scale samples need to be selected for experiments in future studies, and the breadth of sample sources, such as different occupations and different regions, needs to be increased. In terms of assessment, more scientific assessment scales should be used. Meanwhile, the diversity of intervention methods should be increased, so as to further optimize the research effect.

## Author contributions

**Conceptualization:** Hui Han, Chunhua Chen, Rong Sheng, Shiying Wang

**Formal analysis:** Hui Han, Rong Sheng, Shiying Wang

**Funding acquisition:** Hui Han

**Investigation:** Hui Han, Chunhua Chen, Rong Sheng, Shiying Wang

**Methodology:** Hui Han, Chunhua Chen, Rong Sheng, Shiying Wang

**Writing – original draft:** Hui Han, Shiying Wang

**Writing – review & editing:** Hui Han, Rong Sheng, Shiying Wang

**Data curation:** Chunhua Chen, Rong Sheng, Shiying Wang

**Validation:** Chunhua Chen
